# The Pre-ECPR score for predicting favorable neurological outcomes after extracorporeal cardiopulmonary resuscitation: protocol for external validation in the Prague OHCA trial cohort

**DOI:** 10.1016/j.resplu.2025.101213

**Published:** 2025-12-31

**Authors:** Bengt Redfors, Radim Špaček, Anna Henningsson, Lukas Lannemyr, Petra Kmoníčková, Josef Holub, Henrik Imberg, Jan Belohlavek

**Affiliations:** a Department of Cardiothoracic Anesthesia and Intensive Care, Sahlgrenska University Hospital, Gothenburg, Region Västra Götaland, Sweden; b Department of Anesthesiology and Intensive Care Medicine, Sahlgrenska Academy, University of Gothenburg, Gothenburg, Sweden; c Department of Cardiology, Hospital AGEL-Trinec Podlesi a.s., Trinec, Czech Republic; d 3rd Faculty of Medicine, Charles University, Prague, Czech Republic; e 2nd Department of Internal Medicine, Cardiovascular Medicine, General University Hospital and 1st Faculty of Medicine, Charles University, Prague, Czech Republic; f Statistiska Konsultgruppen Sweden, Gothenburg, Sweden; g Department of Molecular and Clinical Medicine, Institute of Medicine, Sahlgrenska Academy, University of Gothenburg, Gothenburg, Sweden; h Institute for Heart Diseases, Wroclaw Medical University, Wrocław, Poland

**Keywords:** Cardiopulmonary resuscitation, Extracorporeal membrane oxygenation, Heart arrest, Patient selection, Predictive value of tests, Reproducibility of results, Treatment outcome, Validation studies as topic

## Abstract

**Aim:**

No clear consensus exists for extracorporeal cardiopulmonary resuscitation (ECPR) patient selection in refractory cardiac arrest. Evidence suggests that current binary criteria yield either low favorable outcome rates, when being liberal, or exclude potential survivors, when being restrictive. The Pre-ECPR score, which uses logistic regression to give a single predicted probability of favorable outcome (modified Rankin Scale 0–3), demonstrated significantly better discriminatory ability than the Extracorporeal Life Support Organization “Example of selection criteria for ECPR”. This protocol outlines the methodology for external validation of the Pre-ECPR score using the ECPR-treated patients from the randomized Prague Out-of-Hospital Cardiac Arrest (OHCA) trial.

**Methods:**

The validation cohort comprises the 92 ECPR-treated patients of the Prague OHCA trial. The Pre-ECPR score incorporates eight predictors: age, no-flow time/initial rhythm combination, total cardiac arrest duration, signs of life, pupil dilation, end-tidal CO_2_, regional cerebral oxygen saturation, and arterial pH, all measured during resuscitation. Model performance will be evaluated through calibration (calibration-in-the-large, calibration slope, and calibration plots); discrimination (area under the receiver operating characteristics curve and Tjur’s discrimination index); and risk stratification analysis at probability thresholds. If needed, model recalibration or refitting will be performed, with internal validation based on k-fold cross-validation. Outcome probabilities will be calculated blinded to study outcomes.

**Conclusion:**

This external validation will indicate whether the Pre-ECPR score retains its predictive performance in an independent OHCA population. The results will provide essential evidence for its potential to improve ECPR patient selection beyond conventional binary selection criteria.

## Highlights

• Protocol for external validation of Pre-ECPR score for patient selection in ECPR.
• In development, the score achieved AUC 0.79 vs 0.63 for ELSO selection criteria.
• Prague OHCA-trial provides an ideal external validation cohort: 92 ECPR patients.
• Prospective data with all physiological predictors systematically collected.
• We present a pre-specified validation framework with blinded outcome assessment.

## Introduction

Cardiac arrest remains a major public health challenge, and despite optimization of conventional cardiopulmonary resuscitation (CPR), many patients fail to achieve return of spontaneous circulation.^1^ Extracorporeal CPR (ECPR) has emerged as a promising intervention for selected patients with refractory cardiac arrest, and American Heart Association guidelines state that the use of ECPR for patients with cardiac arrest refractory to standard advanced cardiac life support (ACLS) is reasonable in selected patients when provided within an appropriately trained and equipped system of care (moderate recommendation with moderate-quality evidence).^2^ Meta-analyses suggest that approximately 20 % of patients treated with ECPR achieve favorable neurological outcomes, defined as a modified Rankin Scale (mRS) of 0–3.^3^, ^4^, ^5^ Consequently, a majority (80 %) of ECPR-patients experience poor outcomes, which is particularly problematic given ECPR's resource-intensive nature.^6^ Optimal selection criteria could minimize futile care while preserving access for those most likely to benefit.^4^

Current selection strategies demonstrate significant heterogeneity, with a systematic review identifying 93 different ECPR selection protocols in the literature.^6^ These protocols typically incorporate age, no-flow time, and low-flow time, and some incorporate physiological parameters such as end-tidal CO_2_ levels or signs of life during CPR.^6^, ^7^, ^8^ The continuous variables are generally converted into binary inclusion or exclusion criteria, with cut-off thresholds varying substantially between protocols.^6^ This dichotomization of continuous parameters represents a major limitation, transforming continuous variables into yes/no criteria with artificial thresholds that fail to capture dynamic relationships with outcomes. Recent retrospective studies demonstrate that restrictive criteria increase survival rates among selected patients but exclude actual survivors who did not fulfill all criteria.^8^, ^9^, ^10^ Liberal criteria, in contrast, exclude few patients, but result in high rates of futile care and low survival rates. Furthermore, evaluation of the patient during ongoing arrest remains limited in most protocols.^4^, ^5^, ^6^ While registry-based prognostic models have been developed, they are limited by available predictors and lack clear ECPR-decision thresholds.^11^, ^12^ As noted in the Extracorporeal Life Support Organization (ELSO) guidelines: “robust data to identify those who may benefit from ECPR are lacking”.^13^

To address these limitations, the Pre-ECPR score was developed using logistic regression to incorporate multiple weighted predictors as continuous variables, predicting the probability of favorable neurological outcomes at one year.^10^ The model incorporates standard predictors and a comprehensive evaluation of circulatory and neurological status during active resuscitation. In internal cross-validation, the score demonstrated superior discriminative performance compared to the ELSO example of selection criteria for ECPR (area under the receiver operating characteristic curve [ROC AUC] 0.79 vs. 0.63, *P* = 0.012).^10^, ^13^ A threshold yielding 100 % sensitivity and 44 % specificity for favorable outcomes was identified. However, external validation is essential to assess the score's generalization performance and reproducibility across different healthcare settings.^14^ The Prague Out-of-Hospital Cardiac Arrest (OHCA) trial provides a well-suited cohort for this purpose, comprising 92 ECPR-treated patients in a prospective randomized clinical trial with systematic collection of pre-ECPR variables and standardized neurological outcome assessments.^15^

This protocol paper outlines the planned methodology for external validation of the Pre-ECPR score, following the framework for external validation by Debray et al.^14^ and adhering to the Transparent Reporting of a multivariable prediction model for Individual Prognosis Or Diagnosis prediction model + AI (TRIPOD + AI) guidelines.^16^ We describe our approach to evaluating the model's predictive performance and validating previously identified decision-making thresholds through blinded probability calculations and cohort comparisons, thereby establishing a methodological foundation before conducting the validation analysis.

## Methods

Ethical approval was obtained from the Regional Ethics Committee of Gothenburg and the Swedish Ethical Review Authority (Dnr 288-17 and 2025-00620-02) and the institutional review board of the General University Hospital and First Faculty of Medicine, Charles University in Prague (192/11S-IV). The study was registered at ClinicalTrials.gov (NCT04198792).

### Validation cohort

**Fig. 1:**
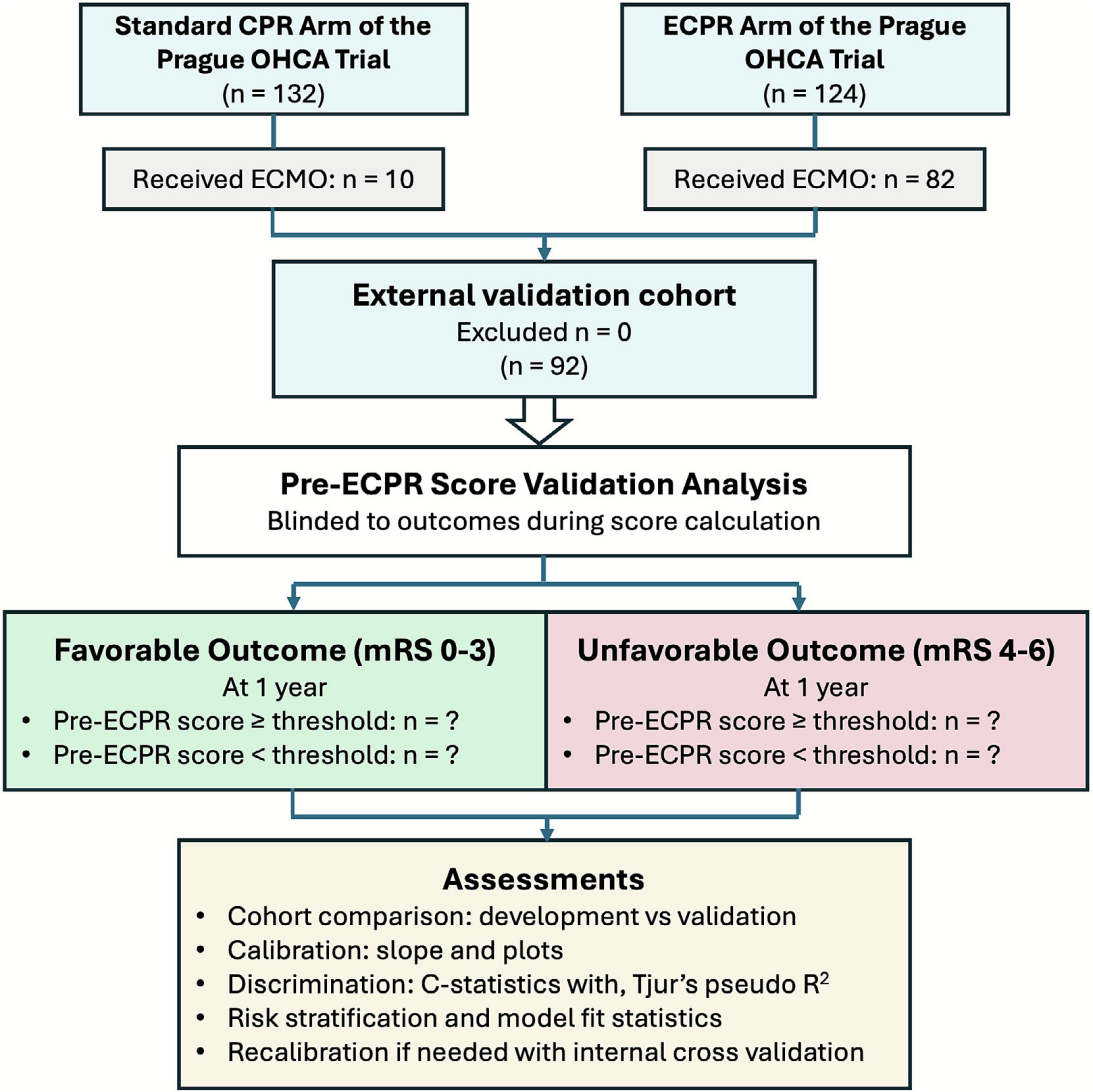
**Flow diagram of the external validation of the Pre-ECPR score using data from the Prague OHCA trial**. A total of 92 ECMO patients will be included in the validation cohort: 82 from the ECPR arm and 10 who crossed over from the standard CPR arm. Patients will be stratified by neurological outcome at one year (favorable: mRS 0–3; unfavorable: mRS 4–6) and by Pre-ECPR score above or below various threshold levels, including the previously identified 6.4% probability cut-off. CPR, cardiopulmonary resuscitation; ECMO, extracorporeal membrane oxygenation; ECPR, extracorporeal cardiopulmonary resuscitation; mRS, modified Rankin Scale; OHCA, out-of-hospital cardiac arrest.

The validation cohort consists of 92 ECPR patients from the Prague OHCA trial, a single-center randomized controlled trial conducted in Prague between March 2013 and October 2020.^15^ The cohort includes adults aged above 18 years who received ongoing resuscitation for witnessed refractory OHCA of presumed cardiac etiology, with inclusion and exclusion criteria detailed in Table 1. Patients were randomized to either invasive strategy (immediate transport for ECPR) or standard strategy (continued on-site CPR). Some patients initially randomized to the standard strategy subsequently crossed over to receive ECPR and are included in this validation cohort, flow diagram in Fig. 1.

**Table 1:** Inclusion and exclusion criteria for the validation cohort, adapted from the Prague OHCA trial.

**Inclusion criteria**	**Exclusion criteria**
**Entry criteria for enrollment into the Prague OHCA trial**	
Age ≥18 and ≤65 years^a^	OHCA of presumed non-cardiac cause
Witnessed OHCA of presumed cardiac cause	Unwitnessed collapse
Minimum of 5 min of ACLS performed by the EMS team without sustained ROSC	Suspected or confirmed pregnancy
Unconsciousness^b^	ROSC within 5 min of ACLS performed by EMS team
ECLS team and ICU bed capacity in cardiac center available	Conscious patient
	Known bleeding diathesis or suspected/confirmed acute or recent intracranial bleeding
	Suspected or confirmed acute stroke
	Known severe chronic organ dysfunction or other limitations in therapy
	“Do not resuscitate” order or other circumstances making 180-day survival unlikely
	Known pre-arrest cerebral performance category (CPC) ≥ 3

**Criteria for initiation of ECLS in the invasive strategy group of the Prague OHCA trial**	
No return of spontaneous circulation or return of spontaneous circulation with ongoing shock (defined as sustained hypotension below 90 mmHg of systolic pressure or need for moderate to high doses of vasopressors)	Signs of death or irreversible organ damage
Admission to cathlab not later than 60 min after the collapse/initial call to emergency medical service^c^	Known bleeding diathesis
Consensus of cardiac center team members on extracorporeal life support initiation	Inadequate arterial and/or venous access for femoro-femoral cannulation

Abbreviations: ACLS: advanced cardiac life support; CPC, cerebral performance category; ECLS, extracorporeal life support; EMS, emergency medical services; ICU, intensive care unit; OHCA, out-of hospital cardiac arrest; ROSC, return of spontaneous circulation.

a Age was estimated at the scene of cardiac arrest; patients later found to be older than 65 years were retained in the study.

b Defined as no response to verbal or painful stimuli during ACLS.

c If collapse time was not exactly known, initial call to emergency medical service was considered.

Patients in the invasive strategy group received mechanical chest compression (LUCAS, Lund University Cardiac Arrest System; Physio-Control Inc/Jolife AB, Lund, Sweden) initiated on scene and were immediately transported to the cardiac catheterization laboratory during ongoing CPR. Drug administration, defibrillations, and other interventions during transport followed European Resuscitation Council guidelines.^17^, ^18^ Intra-arrest intranasal evaporative cooling via a RhinoChill (BeneChill Inc, San Diego, California, USA) device was used when feasible until its discontinuation in 2016.

Patients in the standard strategy group received continued advanced cardiac life support on site according to European Resuscitation Council guidelines. Mechanical chest compression device use was at physician discretion. Ten patients in this group subsequently received ECPR after crossover to the invasive strategy.

Extra corporeal membrane oxygenation (ECMO) cannulation was performed at the catheterization laboratory during ongoing mechanical CPR using a femoro-femoral approach. Following ECMO initiation, patients underwent diagnostic and therapeutic procedures including coronary angiography and percutaneous coronary intervention when indicated. An antegrade perfusion cannula was implanted in the cannulated limb under ultrasound guidance. Continuous anticoagulation with heparin was administered targeting an activated partial thromboplastin time of 50–70 s. All patients underwent immediate biochemical evaluation, urgent bedside echocardiography, and whole-body computed tomography when clinically indicated. Target temperature management to 33 °C was initiated using either the ECMO heat exchanger or other cooling devices. Following publication of updated trial results, temperature management to 36 °C was permitted in cases with early awakening or hypothermia-related complications.^19^ All other post-arrest critical care management, including withdrawal of life-sustaining therapy decisions, followed European Resuscitation Council guidelines.^17^, ^18^

### Outcome

The primary endpoint of the validation is 1-year survival with favorable neurological outcomes, defined as mRS 0–3, consistent with the definition used in Pre-ECPR score development.

In the validation cohort, a neurologist blinded to the initial treatment strategy assessed neurological outcomes at two timepoints: at 6 months after ECPR treatment using the cerebral performance category (CPC) scale, and at a median of 5.3 (interquartile range 3.8–7.2) years using both CPC and mRS scales. To determine 1-year mRS scores, medical records will be systematically reviewed for any intervening events that may have altered neurological status.

### Blinding

The probability of a favorable outcome will be calculated for each individual in the validation cohort while blinded to outcome data. Similarly, comparisons between the model development cohort and validation cohort will be performed blinded to the outcome. Thereafter, outcomes will be unblinded to enable full model performance evaluation and subsequent analyses.

### Prediction model

**Fig. 2:**
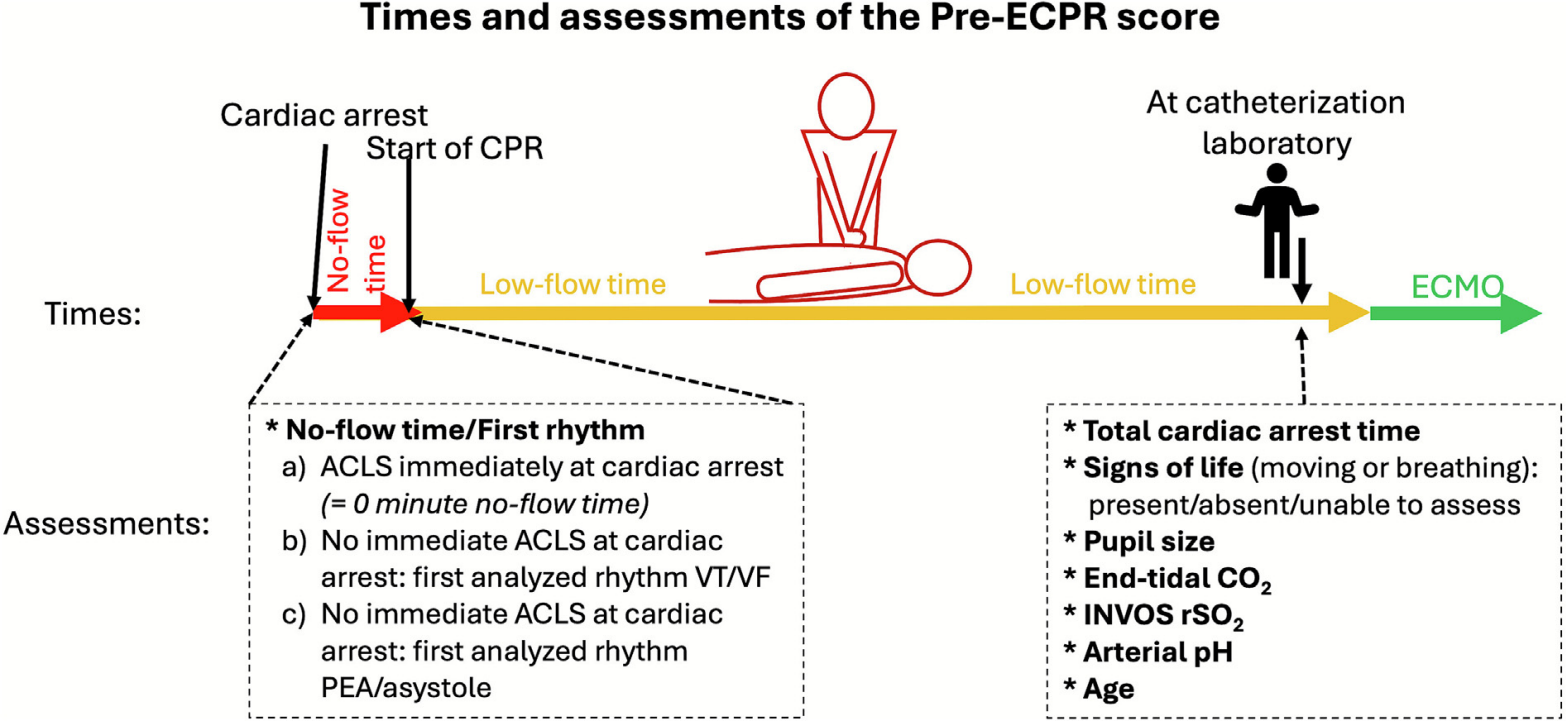
**Assessment points of the Pre-ECPR score**. *Asterisks indicate the eight predictors of the Pre-ECPR score. The composite no-flow/initial-rhythm variable with three levels is determined at cardiac-arrest onset. During CPR, at the time of the ECMO decision, age and patient status are assessed. Signs of life (spontaneous movements or breathing) are classified as present, absent, or unable to assess due to sedation or muscle-relaxant administration. In addition, pupil size, end-tidal CO_2_, INVOS rSO_2_, and arterial pH are assessed. ACLS, advanced cardiac life support; CPR, cardiopulmonary resuscitation; ECMO, extracorporeal membrane oxygenation; INVOS rSO_2_, regional cerebral oxygen saturation measured by near-infrared spectroscopy; PEA, pulseless electrical activity; VF, ventricular fibrillation; VT, ventricular tachycardia.

The Pre-ECPR score estimates the probability of favorable neurological outcomes, defined as an mRS score of 0–3, one year after ECPR.^10^ The model incorporates eight prognostic factors, with five of these being measurements or evaluations performed during the arrest (Fig. 2). The Pre-ECPR score is calculated using a logistic regression model, in which each factor is weighted by its corresponding regression coefficient and contributes to the overall predicted probability of favorable neurological outcomes. Factors include age and total cardiac arrest time (defined as the interval from cardiac arrest onset to ECMO flow initiation). The no-flow time, initial rhythm, and first CPR provider are merged into one variable (no-flow/initial rhythm) with three categories. The first category, “ACLS by medical providers immediately at the cardiac arrest”, refers to situations when medical providers are present at the time of cardiac arrest and initiate ACLS at the moment of the arrest. The two “no immediate ACLS” categories refer to situations in which no ACLS team is present at the time of cardiac arrest. Bystander CPR may have been provided, but the initial rhythm detected later by the ACLS team reflects the degree of anoxic injury sustained by the heart, brain, and body during the no-flow period. These categories are: “No immediate ACLS and first detected rhythm ventricular fibrillation or tachycardia (VT/VF)” and “No immediate ACLS and first detected rhythm pulseless electrical activity (PEA)/asystole”. The former typically indicates less anoxic injury than the latter. Variables assessed at the time of ECMO decision include pupil size, end-tidal CO_2_, signs of life, regional cerebral oxygen saturation (rSO_2_) measured using INVOS 5100 (Medtronic, Minneapolis, MN, USA), and arterial pH closest to the time of ECMO initiation. Signs of life, defined as spontaneous movements or breathing efforts, are categorized into three levels: (a) present, (b) absent, or (c) unable to assess due to sedation or muscle relaxant administration. Measurements of cerebral rSO_2_ are not transferable between brands in cardiac arrest patients; therefore, only INVOS-measured rSO_2_ can be used in the score.^20^ A modified version of the score, excluding INVOS rSO_2_ or arterial pH when missing, is also available; see Table 2 for corresponding formulas. The score is applicable to both in-hospital cardiac arrests (IHCA) and OHCA patients. A Pre-ECPR score calculator is available at ecprscore.org.

**Table 2:** Model specification and regression coefficients for full and modified Pre-ECPR scores.

**Variables**	**Full Pre-ECPR score** **ß coefficient**	**Pre-ECPR score** **without arterial pH** **ß coefficient**	**Pre-ECPR score** **without INVOS rSO**_**2**_ **ß coefficient**
Intercept	**–9.791**	**–6.404**	**–8.827**
Ln (85 – age) [years]	1.593	1.360	1.543
**No-flow time/first rhythm**			
Direct ACLS at CA	0.000	0.000	0.000
No direct ACLS, first rhythm VT/VF	–0.505	–0.514	–0.278
No direct ACLS, first rhythm PEA/asystole	–4.000	–4.000	–4.000
Ln (CA-time) [min]	–0.304	–0.334	–0.324
**Signs of life**			
Not moving or breathing	0.000	0.000	0.000
Not moving or breathing, but sedation or muscle relaxant administered	0.772	0.782	0.798
Moving or breathing	1.528	1.304	1.449
Pupils ≥ 6 mm	–3.916	–3.840	−3.880
Ln (End-tidal CO_2_) [kPa]	0.471	0.693	0.640
Ln (INVOS rSO_2_-14)	0.505	0.513	NA
Ln (Arterial pH-5)	3.799	NA	3.980

Prediction formula (full model): *P*(favorable outcome) = 1/(1 + exp(−LP)), where LP = −9.791 + 1.593 × ln(85-age) − 0.505 if VT/VF − 4.000 if PEA/asystole − 0.304 × ln(CA-time) + 0.772 if sedation given + 1.528 if moving/breathing – 3.916 if pupils ≥ 6 mm + 0.471 × ln(ETCO_2_) + 0.505 × ln(rSO_2_-14) + 3.799 × ln(pH-5). Model constraints: age ≤ 85 years; ETCO_2_ ≥ 1 kPa; rSO_2_ ≥ 15 % (device minimum); pH ≥ 6.

The Pre-ECPR score calculator is available at ecprscore.org.

Abbreviations: ACLS, advanced cardiac life support; CA, cardiac arrest; ECPR, extracorporeal cardiopulmonary resuscitation; ETCO_2_, End-tidal CO_2_; NA, not applicable; PEA, pulseless electrical activity; rSO_2_, regional cerebral oxygen saturation; VT/VF, ventricular tachycardia or ventricular fibrillation.

In the original publication, the Pre-ECPR score demonstrated an ROC AUC of 0.79 (95 % confidence interval [CI] 0.67–0.88) in five-fold internal cross-validation.^10^ No patients with predicted probabilities below 6.4 % achieved favorable outcomes. Above this threshold, the score demonstrated a specificity of 44 % and a positive predictive value of 40.5 % for favorable outcomes. The probability thresholds for 97 % and 94 % sensitivities of favorable outcomes were 12.6 % and 16.0 %, respectively.

### Predictor assessment

All Pre-ECPR score variables were prospectively collected in the Prague OHCA trial, but none was used for decision making regarding ECPR during the study. Age, initial rhythm, no-flow time, and total cardiac arrest time were recorded in the trial's Case Report Form. During patient evaluation in the catheterization laboratory, the following variables were also retrieved: signs of life (spontaneous movements or breathing efforts), pupil size by best visual estimate, end-tidal CO_2_, rSO_2_ using INVOS monitoring, and arterial pH.

### Sample size

With 92 participants and 20 events of favorable neurological outcome, corresponding to an event rate of 22 %, the study is expected to yield the following precision estimates under the assumption of a ROC AUC of 0.80: a 95 % confidence interval halfwidth of approximately 0.12 for the AUC, 0.34 for the observed-to-expected (O:E) event ratio, and 0.7 for the calibration slope.^21^ Calculations were performed using the pmvalsampsize package (version 0.1.0) in R (version 4.2.3).

### Statistical analysis

Model performance will be evaluated in line with established methodological frameworks for external validation of prediction models by Debray et al.^14^

The relatedness of the development and validation cohorts will be evaluated by comparing predictor and outcome frequencies between cohorts, using percentages for categorical variables and means with standard deviations or medians and interquartile ranges for continuous variables, as appropriate. Formal comparisons will be conducted using Pearson’s chi-square test for categorical variables and Welch’s *t*-test or the Mann–Whitney *U* test for continuous variables, depending on data distribution. In addition, c-statistics (AUCs) from logistic regression models predicting cohort membership will be calculated, where a high c-statistic indicates substantial differences between cohorts and a low c-statistic suggests strong similarity. Differences in the distribution of predicted probabilities between cohorts will also be assessed, visualized using box- and scatterplots, and formally tested using Welch’s *t*-test.

Assessment of prediction model performance on the validation cohort will be performed through calibration, discrimination, risk stratification, and model fit statistics. Calibration will be evaluated using calibration-in-the-large, calibration slope, and calibration plots. Calibration-in-the-large assesses whether the predicted probabilities on average are too low, too high, or appropriate. The calibration slope evaluates underfitting or overfitting, that is, whether the predicted probabilities are too close to the mean (underfitting) or too extreme (overfitting), or whether they are adequate. Calibration plots provide a visual presentation of the agreement between observed and predicted event rates. Discrimination ability will be quantified using the ROC AUC and Tjur's discrimination index for the validation cohort, based on development cohort probabilities. A 95 % confidence interval for the AUC will be calculated using the method of DeLong, and discrimination will also be assessed graphically using ROC curves. Risk stratification will be evaluated by calculating sensitivity, specificity, positive and negative predictive values, and positive and negative likelihood ratios at various threshold levels, including the previously identified 6.4 % probability cut-off. McKelvey and Zavoina pseudo-*R*^2^ will be used to assess the model’s explanatory power and goodness of fit.

If the original model demonstrates suboptimal performance or a significant lack of fit in the validation cohort, recalibration will be performed by updating the model intercept and/or calibration slope to align predicted probabilities with observed outcomes. If miscalibration persists or there is evidence of poor discrimination, model refitting may be undertaken, involving re-estimation of model coefficients using the validation data. The performance of the recalibrated or refitted model will then be evaluated through internal cross-validation within the validation cohort to assess the stability and generalizability of calibration estimates.

The Pre-ECPR score is based on a multivariable logistic regression model incorporating eight predictors. To calculate the individual Pre-ECPR score probabilities in the validation cohort, the full model will be used when all data are present, Table 2. Alternative model equations for cases in which arterial pH or INVOS rSO_2_ is missing are available, and for other missing-data patterns further model equations (2*^k^* submodels) will be derived in the development cohort.^22^ Multiple imputation will be used to compute the c-statistics for cohort membership.

Statistical analyses will be conducted using R software (version 4.4.3 or later; R Foundation for Statistical Computing, Vienna, Austria), and results will be reported in accordance with the TRIPOD + AI guidelines.^16^

## Discussion

Optimal patient selection is essential for effective ECPR implementation. However, current selection protocols, largely based on dichotomous cut-offs, risk excluding salvageable patients and exposing others to futile invasive therapy.^9^ The Pre-ECPR score is the first to merge static variables (age, initial rhythm), continuous arrest duration, bedside physiological markers (pH, rSO_2_, end-tidal CO_2_), and simple clinical observations (pupil size, spontaneous movements) – providing a more nuanced probability estimate than traditional binary criteria.^10^ In internal cross validation, the score showed superior discrimination compared to the ELSO guideline criteria and exceeded registry scores. However, internal validation alone is insufficient to establish the model’s generalization performance across different settings.^14^ Factors such as variations in patient characteristics, clinical practices, and confounders can affect performance. Therefore, external validation in an independent population is an essential next step before broader implementation can be considered. The Prague OHCA trial provides a well-suited validation cohort with prospectively collected data with standardized protocols and systematic variable collection during arrest.^15^

Despite ECPR's potential, evidence to guide optimal candidate selection remains scarce, posing significant challenges in predicting which patients will achieve favorable outcomes, while simultaneously minimizing futile care.^13^ Consequently, a recent systematic review identified 93 different ECPR protocols across 90 studies, highlighting the lack of consensus in patient selection.^6^ Traditional approaches rely on the dichotomization of continuous parameters.^5^, ^6^ These criteria show marked variability: age limits range from 50 to 80 years, no-flow time from 0 to 20 min, and low-flow time from 30 to 150 min.^6^ The most frequently used cut-offs are age 65–75 years, no-flow time 5 min and low-flow time 60–100 min in previous protocols. However, while such dichotomized criteria aim to stratify risk, they inevitably create artificial thresholds that fail to capture the nuanced relationship between continuous prognostic factors and outcomes. Diehl et al. clearly demonstrated this dilemma in a multi-regional Australian dataset, where the restrictive CHEER criteria would have achieved a 44 % survival rate, compared to 22 % with the liberal Prague OHCA criteria.^9^ However, these restrictive criteria would have excluded 63 % of all actual survivors. Similarly, for IHCA, the restrictive CHEER criteria would have excluded 77 % of all ECPR survivors. The same pattern was shown in the development cohort of the Pre-ECPR score, where the ELSO example of selection criteria for ECPR missed 22 % of the patients with favorable outcomes.^10^

Furthermore, although anoxic brain injury is the most common cause of poor ECPR outcomes, only 30 % of protocols include physiological parameters during cardiac arrest, most commonly an end-tidal CO_2_ cut-off of 1.33 kPa (10 mmHg).^6^ Yet evidence demonstrates that severely dilated pupils predict extremely poor survival, low cerebral rSO_2_ during arrest independently correlates with unfavorable outcomes, and among OHCA survivors, most exhibit signs of life during resuscitation.^23^, ^24^, ^25^, ^26^ These findings underscore the prognostic value of real-time physiological assessments during cardiac arrest – assessments that remain underutilized or reduced to cut-offs in traditional protocols.

In contrast to previous protocols, the Pre-ECPR score offers a comprehensive evaluation by incorporating both the standard parameters (age, initial rhythm, and arrest times) and a real-time assessment of the patient during cardiac arrest, including signs of life, pupil dilatation, end-tidal CO_2_, INVOS rSO_2_, and arterial pH. These variables are used as continuous rather than dichotomized parameters, and they are weighted together by logistic regression to provide a single, unified prediction of the probability of favorable outcomes at one year, thus using the full predictive power of each parameter. The Pre-ECPR score identified a clinically meaningful threshold: 40 % of patients above this threshold achieved favorable outcomes, while none below it survived with favorable neurological function.

The Prague OHCA trial provides a high-quality validation cohort for several reasons. As the largest randomized CPR/ECPR trial to date, it offers prospectively collected data with standardized protocols and systematic assessment of all variables required for the Pre-ECPR score during cardiac arrest. The trial's design ensures high-grade data collection and limited missing variables. Furthermore, outcome assessment by neurologists ensures reliable and standardized neurological outcome classification. Beyond these trial characteristics, the validation methodology follows Debray's framework for external validation and adheres to TRIPOD + AI guidelines, with blinded outcome assessment to minimize bias.^14^, ^16^

Several limitations warrant consideration. Although the Prague OHCA trial represents the largest randomized ECPR trial to date with high-quality prospective data, the validation cohort of 92 patients with 20 events limits statistical precision.^21^ While the overall validity of the Pre-ECPR score model can be assessed, the sample size constrains the ability to detect subtle miscalibration or diminished discriminative performance. Larger ECPR cohorts are available in registries; however, these lack the detailed physiological assessments during cardiac arrest that are essential for calculating the Pre-ECPR score.^11^, ^12^ This external validation constitutes an important step in testing the score's generalizability and identifying potential needs for recalibration, but additional validation studies and regulatory approvals – including Conformité Européenne marking for Europe and Food and Drug Administration approval for the United States – will be required before broader clinical implementation can be considered. The Pre-ECPR score was developed for both IHCA and OHCA populations, but the present validation cohort includes only OHCA patients. Thus, this study will specifically validate the score's performance in the OHCA population. The use of intra-arrest cooling with RhinoChill in some patients could influence neurological status at ECPR assessment, particularly signs of life, requiring careful consideration during data analysis. Lastly, variations in post-ECPR care protocols across centers might influence outcomes and should be acknowledged as a potential source of heterogeneity.

## Conclusion

This protocol outlines a pre-specified and transparent plan for external validation of the Pre-ECPR score using data from the Prague OHCA-trial, a high-quality randomized clinical trial. The analysis will adhere to state-of-the-art methodological standards for external validation and be reported in accordance with the TRIPOD + AI guidelines. The findings will determine whether the predictive performance demonstrated in internal validation is preserved in an external validation cohort, indicating the potential for broader clinical applicability.

## Declaration of generative AI and AI-assisted technologies in the writing process

During the preparation of this work the authors used Claude/Anthropic in order to improve language and readability. After using this tool, the authors reviewed and edited the content as needed and take full responsibility for the content of the published article.

## CRediT authorship contribution statement

**Bengt Redfors:** Writing – review & editing, Writing – original draft, Methodology, Funding acquisition, Conceptualization. **Radim Špaček:** Writing – review & editing. **Anna Henningsson:** Writing – review & editing. **Lukas Lannemyr:** Writing – review & editing. **Petra Kmoníčková:** Writing – review & editing. **Josef Holub:** Writing – review & editing. **Henrik Imberg:** Writing – review & editing, Methodology. **Jan Belohlavek:** Writing – review & editing, Supervision, Conceptualization.

## Declaration of competing interest

The authors declare the following financial interests/personal relationships which may be considered as potential competing interests: Bengt Redfors reports financial support was provided by The Swedish Herat-Lung foundation. Bengt Redfors reports financial support was provided by the Swedish state under the agreement between The Swedish Government and the county councils, the ALF- agreement. Jan Belohlavek reports a relationship with Abiomed/JMJ Medtech that includes: speaking and lecture fees, relationship with Getinge AB that includes: speaking and lecture fees, Astra-Zeneca that includes: speaking and lecture fees and with Boegringer-Ingelheim that includes: speaking and lecture fees. If there are other authors, they declare that they have no known competing financial interests or personal relationships that could have appeared to influence the work reported in this paper.
